# Evaluation of the rupture of silicone breast implants by mammography, ultrasonography and magnetic resonance imaging in asymptomatic patients: correlation with surgical findings

**DOI:** 10.1590/S1516-31802004000200002

**Published:** 2004-03-01

**Authors:** Anabel Medeiros Scaranelo, Américo Ferreira Marques, Elizabeth Brenda Smialowski, Henriquel Manoel Lederman

**Keywords:** Silicone, Breast implants, Rupture, Mammography, Ultrasonography, Magnetic resonance imaging, Ruptura, Mamografia, Ultrasonografia, Ressonância magnética, Cirurgia plástica, Mamoplastia

## Abstract

**CONTEXT:**

Different imaging methods can identify the integrity of breast implants and also the extent of possible silicone leakage. Mammography, ultrasonography and magnetic resonance imaging are often used to evaluate the integrity of breast implants, usually in patients that are symptomatic for rupture. A group of clinically asymptomatic patients was taken as a sample. These patients wanted to remove or change their breast implants for psychological or cosmetic reasons.

**OBJECTIVE:**

The purpose of this study was to compare the efficacy of mammography, sonography and magnetic resonance imaging in the detection of breast implant rupture in an asymptomatic population.

**TYPE OF STUDY:**

Prospective study.

**SETTING:**

Department of Diagnostic Imaging, Universidade de São Paulo, São Paulo, Brazil.

**METHODS:**

The participants were 44 asymptomatic patients who subsequently had implants surgically removed. Eighty-three implants were evaluated by both film-screen mammography and high-resolution sonography and 77 implants were evaluated by magnetic resonance imaging. The sensitivity and specificity of mammography, ultrasonography and magnetic resonance imaging were assessed using predetermined diagnostic criteria for implant rupture. All radiological signs were discussed and false positives and false negatives were retrospectively evaluated to identify the pitfalls in the investigations.

**RESULTS:**

The respective sensitivity and specificity of mammography were 20% and 89%; sonography, 30% and 81%; and magnetic resonance imaging, 64% and 77%. The differences between patients with breast implants for cosmetic and oncological reasons were discussed.

**CONCLUSIONS:**

Our experience suggests that magnetic resonance imaging seems to be the best imaging method on its own for the evaluation of rupturing among asymptomatic patients.

## RESUMO

**CONTEXTO:**

Métodos diferentes de imagem podem identificar a integridade das próteses mamárias e também a extensão de um possível extravasamento do silicone. Mamografia, ultra-sonografia e ressonância magnética são comumente utilizados para avaliar a possibilidade de ruptura de próteses em pacientes que são sintomáticos. Um grupo de pacientes clinicamente assintomáticas para ruptura foi tomado como amostra nesse trabalho. Elas estavam descontentes com suas próteses e queriam removê-las ou trocá-las por razões psicológicas ou estéticas.

**OBJETIVO:**

O propósito deste trabalho foi comparar a eficácia da mamografia, ultra- sonografia e ressonância magnética na detecção de ruptura de prótese mamária numa população assintomática.

**TIPO DE ESTUDO:**

Trabalho prospectivo.

**LOCAL:**

Departamento de Diagnóstico por Imagem, Universidade Federal de São Paulo/Es-cola Paulista de Medicina, São Paulo.

**MÉTODOS:**

44 pacientes assintomáticas para ruptura que tiveram suas inclusões removidas cirurgicamente. Foram realizadas mamografia e ultra-sonografia de 83 próteses e ressonância magnética de 77 próteses. Sensibilidade e especificidade da mamografia, ultra-sonografia e ressonância magnética utilizando critérios diagnósticos predeterminados para ruptura. Os sinais radiológicos encontrados são discutidos e os falsos positivos e negativos são retrospectivamente avaliados para identificar quais foram as falhas diagnósticas, utilizando-se os achados operatórios como padrão ouro.

**RESULTADOS:**

A sensibilidade e especificidade da mamografia foram de 20% e 89%, respectivamente; da ultra-sonografia, de 30% e 81%, respectivamente, e, da ressonância magnética, de 64% e 77%. Ressaltam-se as diferenças apresentadas pelos grupos: próteses incluídas por motivo estético e por motivo oncológico.

**CONCLUSÕES:**

Concluímos que a ressonância magnética é o método preferencial isolado a ser utilizado.

## INTRODUCTION

Silicone gel implants are used for reconstructing breasts after mastectomy, correcting congenital or traumatic deformities and increasing or remodeling breast shape for cosmetic reasons. The psychological benefits of this procedure are widely acknowledged. However, silicone breast implants can lead to a range of significant complications such as hardening and rupture.^1^

It is very difficult to make a preoperative diagnosis of rupturing from physical examination because the symptoms and signs are usually vague, especially when the rupture is enclosed by the fibrous capsule.^2,3^ Different imaging methods can identify the integrity of the breast implants and also the extent of possible silicone leakage going to the glands and adjacent tissues. Mammography, ultrasonography, computerized tomography and magnetic resonance imaging have been used to evaluate the integrity of breast implants in symptomatic patients, in relation to rupturing.^4-6^

In order to evaluate the performance of three different types of diagnostic imaging method (mammography, ultrasonography and magnetic resonance imaging) in the detection of silicone breast implant rupturing in asymptomatic patients, some signs were analyzed and compared with the surgical specimens.

## METHODS

A group of clinically asymptomatic patients was taken as a group sample. These patients wanted to remove or change their breast implants for psychological or cosmetic reasons.

There were a total of 83 silicone gel breast implants among 44 patients referred to the Department of Diagnostic Imaging of the Universidade Federal de São Paulo — Escola Paulista de Medicina for evaluation through mammography, ultrasonography (US) and magnetic resonance (MR) imaging, during the period from December 1993 to November 1996.

The patients examined were unhappy with the esthetic appearance or hardening of their breasts. They did not present any breast lump during the physical exam or any decrease in implant volume during the preoperative evaluation for the surgical correction. They were, thus, considered clinically asymptomatic in relation to implant rupture. Patients who presented a history of change in their implants were excluded from this study.

The clinical information collected included the time of implant inclusion (for the calculation of the age of the material), the position (subpectoral or prepectoral), the reason for the inclusion (cosmetic or oncological) and the implant type (silicone whole gel, also referred to as single lumen).

All 44 patients were submitted to mammography and ultrasonography. Magnetic resonance imaging was not performed on three patients, due to claustrophobia.

Mammography was performed using the routine oblique mediolateral, craniocaudal and implant displacement views (600 T, 0.3 mm machine; General Electric Medical Systems, Milwaukee, Wisconsin). Ultrasonography was done using linear multifrequency 7.5-10 MHz real time transducers in the Ultramark 9 HDI equipment (ATL – Advanced Technology Laboratories, Bothell, Washington). For the evaluation of the posterior walls of the implant and for very large breasts, a sectoral probe of frequency 3.5 - 3.75 MHz was also used. Magnetic resonance imaging studies were performed via either the Philips 1.5 T Gyroscan MR scanner (Philips Medical Systems, Netherlands), to evaluate 57 implants, or the GE 0.5 T Sigma Advantage MR scanner with V.5.4 software (General Electric Medical Systems, Milwaukee, Wisconsin), to evaluate 20 implants. Different coils were used: a body coil in the 1.5 T equipment, and a dedicated breast coil in the 0.5 T equipment.

The scan protocol in the 1.5 T MR scanner consisted of T2-weighted SE axial and sagittal images (2000/80) and T1-weighted FSE axial images (750/20); 5 to 6 mm slice thickness; and 256 × 180 acquisition matrix. The following parameters were used for the 0.5 T magnet: T2-weighted FSE axial and sagittal images (4000/170); 256 × 256 acquisition matrix, and 3.6 mm slice thickness. No intravascular contrast agent was used, nor was cardiac or respiratory gating used, because they would have increased the acquisition time.

Results of each mammography, sonography and magnetic resonance imaging were independently analyzed by a single investigator (A.M.S.) who was unaware of the results from the other methods. The patient’s identification or name was also concealed from this investigator. For each implant analyzed, a three-category classification system was used: normal, suspicion of rupture, diagnosis of rupture (Table 1).

**Table 1:** Classification system for the rupture of breast implants

Category	Mammography	Ultrasonography	Magnetic resonance imaging
Normal	Regular shape	Anechoic interior	Regular shape
	No curve alteration	Low level echoes	No wavy or discreet curve
	Smooth curve shape	Anterior wall reverberation	Homogeneous gel signal
	Oval or round form	Isolated echogenic line	Elastomeric folds Bubbles in the gel interior
Suspicious	Major bulges	Amorphous internal echoes	Bulged contours
	Loss of round or oval shape	Discontinuous echogenic lines	Loss of round or oval form
		Very thick isolated echogenic line	
Diagnostic	Irregular/ill-defined borders	Stepladder sign	Linguine sign
	Silicone outside of implant	Echogenic noise associated with:	Teardrop sign
	Including granuloma and silicone adenopathy	echolucent globules	Silicone outside of implant
		echodense globules	

The time that elapsed between the imaging examinations and the surgery did not exceed one week.

All patients had their breast implants removed by a single surgeon (A.F.M.) and the alterations found were classified as follows:

### Normal implants:

when the elastomeric envelope was complete, without perforations, but presented an external silicone gel layer at the time of implant removal due to gel bleeding. These implants were considered not to be ruptured, after the elastomer was carefully evaluated by the surgeon in charge of rupture investigation.

### Ruptured implants:

when there was elastomer discontinuity and consequently the spread of silicone into the surrounding tissues. This category includes both extracapsular and intracapsular ruptures, because there was interest in determining rupture and non-rupture in the sample analyzed.

The statistical analysis was done via the chi-squared and Fisher exact tests, for each meaningful finding defined as normal, suspicious or diagnostic. All of these were compared with the surgical results. Following this, the totals of the results found were individually compared to the surgical finding, according to each examination type, after applying the McNemar test. The surgical finding was considered to be the gold standard.

The same procedure was used for the partial results from magnetic resonance imaging. The implant analyses done via the 1.5 T and 0.5 T equipment were put together. Each imaging method was also compared with the surgical finding, and the samples were divided according to the reason for the implant: cosmetic or oncological. The sensitivity of each method was determined by means of the relationship between the true positive values (ruptured implants found during surgery that were correctly diagnosed by the imaging method as ruptured) and the total number of true positive and false negative values (total number of ruptured implants).

The specificity was determined by means of the relationship between the true negative values (non-ruptured implants found during surgery that were correctly diagnosed as such by the imaging method) and the total number of non-ruptured implants in the sample, considering both the true negatives and false positives. The rejection level for the nullity hypothesis was set at 0.05 or 5% (p ≤ 0.05) for all tests. The significant values were marked with an asterisk (*).

The Institutional Review Board of Universidade Federal de São Paulo/Escola Paulista de Medicina (Unifesp/EPM) approved this study.

## RESULTS

Thirty-nine patients presented bilateral implants and five patients presented unilateral implants. The implants had been done for cosmetic reasons in 21 patients (50.6%) and for oncological reasons (due to breast reconstruction after mastectomy) in 23 patients (49.4%). The implant positions in relation to the pectoral muscles were as follows: 65 (78.3%) were located in the subglandular or prepectoral position and 18 (21.7%) were located in the retropectoral position.

Among the 83 implants studied, 30 of them were found to be ruptured (36%) during surgery and 53 non-ruptured (64%). In addition, 27 (32.5%) of these non-ruptured implants already presented gel bleeding and 26 (31.5%) were intact. The average age of the ruptured implants was 11.9 years. On the other hand, the average age of the bleeding implants was 11.7 years, and the intact ones were 11 years old. In relation to the time elapsed since implant inclusion, the rupture rate was 0% between one and five years; 35.29% between six and 10 years; 36.36% between 11 and 15 years; and finally 66.66% between 16 and 18 years.

Through mammography, 71 implants were considered normal and 12 altered (suspicious or diagnostic). Through ultrasonography, 64 implants were considered normal and 19 altered (suspicious or diagnostic). Through magnetic resonance imaging, 49 implants were considered normal and 28 altered (suspicious or diagnostic). Through 1.5 T magnetic resonance imaging (n = 57), 40 implants were considered normal and 17 altered (suspicious or diagnostic). Finally, through 0.5 T magnetic resonance imaging with breast coil (n = 20) 10 implants were considered normal and 10 altered (suspicious or diagnostic).

The sensitivity of mammography was 20% (6 in 30), ultrasonography 64% (16 in 25) and magnetic resonance imaging 64% (16 in 25). Furthermore, the 1.5 T and 0.5 T magnetic resonance sensitivities were respectively 50% (8 in 16) and 80% (8 in 10). The differences found between the different methods were statistically significant (p < 0.05) for the mammography and ultrasonography data, while not significant for the magnetic resonance imaging.

The specificity found was 88.7% (47 in 53) for the mammography, 76.9% (40 in 52) for the ultrasonography and 76.9% (40 in 52) for the magnetic resonance imaging. In addition, the specificity found for the 1.5 T and 0.5 T magnetic resonance imaging was respectively 78% (32 in 41) and 80% (8 in 10). Once again, there was no statistically significant difference for the magnetic resonance data. However, the difference was significant for the mammography and ultrasonography data.

The positive and negative predictive values and likelihood ratios are summarized in Table 2. The 0.5 T magnetic resonance imaging with breast coil presented better sensitivity, specificity, and positive and negative likelihood ratio results.

**Table 2:** Sensitivity, specificity, positive predictive value, negative predictive value and likelihood ratio for mammography, ultrasonography and magnetic resonance imaging

Method(%)	Specificity (%) value (%)	Sensitivity predictive value (%)	Positive predictive (+)	Negative e ratio (-)	Likelihood ratio	Likelihood
Mammography	88.7	20	50	66.2	1.77	0.90
Ultrasonography	81.2	30	47.4	67.2	1.59	0.86
Magnetic resonance imaging	76.9	64	57.1	81.6	2.77	0.47
Magnetic resonance imaging (0.5 T)	80	80	80	80	4.00	0.25
Magnetic resonance imaging (1.5 T)	78	50	47	80	2.28	0.64
Mammography + ultrasonography	88.4	11.8	28.6	71.7	1.01	1.00
Ultrasonography + magnetic resonance imaging	78.7	53.8	41.2	86	2.53	0.59
Magnetic resonance imaging + mammography	94.7	33.3	66.6	81.8	6.33	0.70
Mammography + ultrasonography + magnetic resonance imaging	93.9	22.2	50	81.6	3.67	0.83

These data were also analyzed to determine whether there would be any benefit from the interaction of these methods when combined in pairs or using all three methods together. For this, we used the rule that the implant could only be considered altered if the two or three methods had the same result. It can be seen in Table 2 that the combination of these methods caused a decrease in sensitivity in magnetic resonance imaging when any combination was used, with little improvement of specificity.

## DISCUSSION

Complications after breast implant surgery, particularly implant rupture, that are clinically occult can be detected by imaging methods. This study compared three different imaging tests (mammography, ultrasonography and magnetic resonance imaging) performed in a group of patients who were clinically asymptomatic for rupture but had long implant inclusion times. The objective was to search for the most useful method for detecting implant rupture. The long inclusion times provide the justification for tests to determine the integrity or non-integrity of such implants.

Mammography is an extremely specific method for the diagnosis of extracapsular rupture that can detect silicone gel migration through the glandular parenchyma.^2,7,8-10^ On the other hand, the diagnosis of intracapsular rupture cannot be performed via mammography.^5^ The most important mammographic pattern is the changing of implant shape.^7^ Large bulges may represent focal areas of implant rupture or herniation of an intact implant, leading towards defects in the capsule after a closed capsulotomy or direct trauma.^6^ Bulges were found in this study in practically the same proportions in non-rup- tured and ruptured implants.

Mammograms are good screening tests for the rupture of breast implants because the incidence of false positives is low.^10^ Our study showed 20% sensitivity, 88.7% specificity and a likelihood ratio of 1.77, thus confirming that mammography is not considered to be the choice for intracapsular rupture diagnosis. The more extracapsular-type ruptures there are in the sample, the higher the sensitivity and the predictable positive result of the mammographic method are. In this study, only 3 out of 30 ruptures were extracapsular. Andersen et al.^10^ reported higher sensitivity of mammography (67%) because they included patients with a history of breast trauma. Other authors, including us, did not do this and found sensitivities ranging from 16.2 to 23%.^4,11^

**Figure 1:**
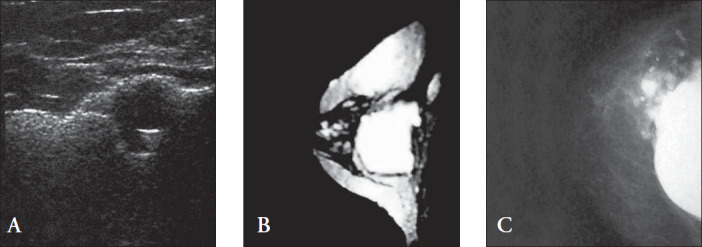
(A) Longitudinal ultrasonogram in the upper lateral quadrant region of the right breast showing an echolucent globule adjacent to snowstorm appearance. (B) Sagittal fast spin echo magnetic resonance image showing localized rupture of the implant in the right breast. (C) Craniocaudal view mammogram showing abnormal implant, thereby demonstrating rupture and extravasation of free silicone into the breast.

Ultrasonography has been used in breast implant integrity evaluation for several years. A great variety of ultrasound signs suggesting rupture have been described.^3-7,12-25^ The most useful of these signs is the "echodense noise" or snowstorm appearance, which shows the occurrence of diffusely increased free silicone echogenicity in the mammary tissues.^15^ This sign (Figure 1) was present in 10% of the ruptured implants. It was statistically significant (p = 0.0441) and similar to other series.^16,24^

**Figure 2:**
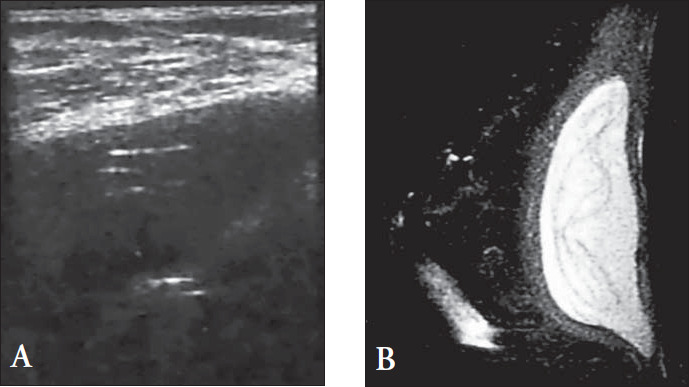
Intracapsular rupture with collapsed elastomer shell. (A) Ultrasonography of contained intracapsular ruptured implant showing multiple layers of the collapsed implant shell visible as sets of parallel echogenic lines (stepladder sign). (B) Sagittal magnetic resonance image showing the same aspect of the collapsed implant shell (linguine sign).

Another ultrasound sign, called the stepladder, has been described as predictive of rupture. This sign corresponds to the linguine sign found in magnetic resonance imaging and is shown in ultrasonography images as a series of parallel horizontal or curved echogenic lines beyond the interior of the implant that correspond to ruptured elastomer.^16^ The stepladder sign (Figure 2) was detected in 16.66% of the ruptured implants and 3.77% of the non-ruptured ones (major atypical folds that simulated the stepladder sign), and the difference was statistically significant (p = 0.0553). The stepladder sign has also been found in severe capsular contracture and it has been suggested that, in the absence of other echographic signs for rupture such as echodense noise, the stepladder sign might not be a diagnostic sign for implant rupture.^23^ However, other authors^16,21,26^ found that the stepladder sign was highly specific for rupture, as we found in the present study.

Although the criteria used for implant integrity evaluation through ultrasonography were statistically significant (p = 0.0354), the data from the present study showed that ultrasonography was not a very sensitive method (30%) in the detection of breast implant ruptures, unlike the findings of other authors (ranging from 70 to 74%).^16,22,23^ The falsenegative and false-positive rates found in the present work were 25% and 12%, respectively, with 30% sensitivity, 81.13% specificity and a likelihood ratio of 1.59. These rates are related to the high prevalence of non-specific echographic signs in both ruptured and nonruptured implants. It was observed that, in studies that included samples that were asymptomatic for rupture, the sensitivity was lower than what was described in the present study.^16,19,22,23^

In our study, ultrasound showed limitations, thereby indicating that in patients with major folds it is extremely difficult to rule out rupture and that this should be investigated through magnetic resonance imaging. Ultrasonography can be used as the first test in the assessment of breast implants in asymptomatic patients, followed by mammography. This is because ultrasonography has greater ability to detect intracapsular rupture types (clinically occult) and, moreover, it has the same sensitivity as magnetic resonance imaging in detecting extracapsular ruptures.

Magnetic resonance imaging has been proposed as a method for silicone implant evaluation because of its high sensitivity and specificity in both intra and extracapsular rupture detection.^4,5,27^ The most revealing sequence in the present investigation of silicone breast implants was the T2 weighted sequence, as also described by other authors.^28,29^

The homogeneous high signal intensity of the silicone gel was considered to be a predictive sign for non-rupture (χ^2^ = 6.634; p < 0.05). This conclusion came from the fact that 46.15% of non-ruptured implants (40.7% of the bleeding ones and 54.1% of the intact ones) presented such signal intensity, in comparison with only 16% of the ruptured ones.

Another common finding in normal implants is the presence of isolated radial folds. Ruptured implants that present this sign are infrequent.^6,30^ In the present study, the presence of isolated radial folds was significant: they were found in 38.5% of the non-ruptured implants and 8% of the ruptured ones. The presence of folds indicates a certain degree of capsular contraction or tense elastomer.

**Figure 3:**
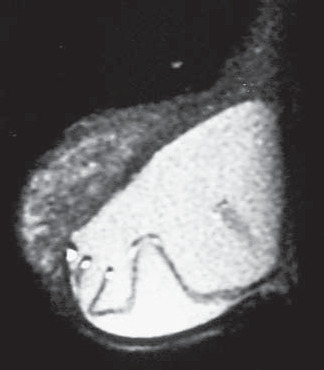
Intact breast silicone implant. Sagittal fast spin echo T2-weighted magnetic resonance image showing atypical folds that simulate linguine appearance of a ruptured implant.

The linguine sign (pasty appearance of fragmented elastomers, as seen on magnetic resonance imaging) was considered to be predictive for intracapsular rupture in the present study. It was found in 64% of the ruptured implants (χ^2^= 17.917; p < 0.05) and in 1.92% (1 in 52) of the non-ruptured ones (Figure 2). The linguine sign was not detected in bleeding implants. The false positives were due to the presence of atypical folds (Figure 3) that mimicked ruptured wavy elastomer. We therefore believe that the use of orthogonal planes with lesser slice thickness, or volumetric acquisition with volumetric reformatting, can minimize such chances of error.

The teardrop sign (entrapment of silicone gel inside a fold, thus mimicking the appearance of an inverted tear) was found in 12% of the ruptured implants and none of the bleeding or intact implants. In contrast, Berg et al.^26^ found this sign in 25% of the ruptured implants, 36% of the bleeding ones and 6% of the intact ones. A possible criticism of the present study is that the low signal-to-noise ratio may be responsible for the poor visualization of this sign. When the signal-to-noise ratio was studied in order to view the linguine sign and characterize the folds, these signs were visible with or without acquisition using the dedicated breast coil.^31^ The teardrop sign may not have adequate spatial resolution because it is more subtle than the linguine sign.

The present false negatives found in magnetic resonance imaging were secondary to the ruptured non-collapsed elastomer located outside the implant, while keeping the homogeneous high signal intensity inside it. The terminology "rupture without collapse" was used by Berg et al.^26^ to explain the envelope adherence to the fibrous capsule phenomenon. This terminology explains the absence of linguine sign and the gel homogeneity from magnetic resonance imaging that were found in some ruptured implants in this study.

Microscopic quantities of silicone may be present outside of the implant as a normal characteristic due to transudation. However, the presence of macroscopic quantities of silicone surpassing the limits of the fibrous capsule has been described by magnetic resonance imaging in only 4.7 to 20% of ruptures, by different authors.^6,26,29,32^ Extracapsular silicone was present in only 10% of surgically proven ruptures in our study. Only two of the three implants with extracapsular rupture were visualized here. The false negative result was due to the presence of blocked rupture, which was misdiagnosed as an irregular contour bulge.

The diagnostic confusion between small normal pleural effusion and extracapsular rupture found via magnetic resonance imaging has been previously described.^33^ In about 20 to 25% of patients, small quantities of pleural liquid may be present posteriorly to the breast on magnetic resonance images. This occurs because of the ventral decubitus position used in most of these procedures. The false positive in the present series was related to the presence of hyperintense material in the T2 weighted images, in deeper juxtacostal and juxtapleural areas, adjacent to bleeding prepectoral implants. These areas were reported to consist of free silicone, but they corresponded to neoplastic exsudate within the pleural space.

**Figure 4:**
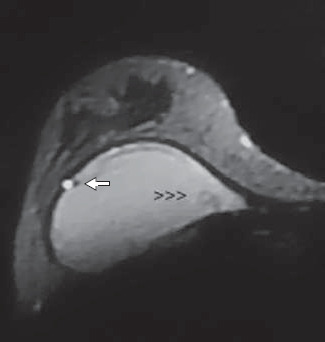
Intact silicone prosthesis with heavy gel bleeding identified at surgery. Axial fast spin echo magnetic resonance image of the right breast showing a small teardrop extending to the medial aspect of the implant (arrowheads) and a hyperintense water droplet *(arrow)* in conjunction with subcapsular lines.

The findings of hyperintense dots inside the gel do not indicate rupture. Some authors have described findings of air bubbles or water droplets within the silicone gel. These air bubbles or water droplets were associated with the linguine sign and ruptured implants.^6,26,28,30,34^ We found hyperintense dots in 7.7% of nonruptured implants. Of these implants, three presented bleeding (Figure 4) and one was intact. The only ruptured implant that presented this sign had the linguine sign at the same time. However, it is important to recognize this sign as an alert in the search for subtle signs of intracapsular rupture. This sign is often associated with the subcapsular line, the linguine sign and the base of the teardrop sign, which are indicative of rupture.^30^

Magnetic resonance imaging performed with the two equipment types showed 64% sensitivity, 76.92% specificity, 42.85% falsepositive, 18.36% false-negative, positive likelihood ratio of 2.77 and negative likelihood ratio of 0.47. Considering each equipment type separately, the 0.5 T magnetic resonance imaging showed 80% sensitivity and specificity and a positive likelihood ratio of 4.00; the 1.5 T magnetic resonance imaging showed 50% sensitivity, 78.04% specificity and a positive likelihood ratio of 2.28.

Our results were less sensitive than some others that had very low false negative rates,^28^ but the latter patients presented with clinical symptoms suggestive of implant rupture. Other authors^3,6^ who included a number of asymptomatic patients in their studies, were unfortunately unable to prove their image findings surgically or could only obtain small samples. However, when we compared some selected parts of the samples, in an attempt to use the same methodology as in criterion III of Reynolds et al.,^6^ which was equivalent to only some of the diagnostic findings from our study, we acquired more sensitive results.

When we evaluated magnetic resonance imaging equipment with body coil and surface coil dedicated to the breast, thereby reproducing the methodology used by Weizer et al.,^3^ we obtained better sensitivity and quite a difference in specificity. This was because these were the last tests performed in our series, and the investigators were already on the second half of the learning curve acquired through this study. Although the number of patients with tests performed on 0.5 T equipment was almost one-third of the number with tests performed on the 1.5 T equipment, the number of false positives and false negatives presented by the 1.5 T equipment was almost four times higher. Statistical evaluation of the tests performed on the 0.5 T and 1.5 T equipment did not show significant differences (p > 0.05), in comparison with the surgical findings.

When the methods were compared with each other in the present study, we found 76.62% agreement of magnetic resonance imaging + ultrasonography and 59.74% agreement of magnetic resonance imaging + mammography. These data show that both magnetic resonance imaging and ultrasonography are methods that allow the detection of implant alterations, in relation to mammography. In addition, the data show that magnetic resonance imaging allows the detection of very many alterations that are considered suspicious or diagnostic by both methods.

The data relating to the agreement of mammography + ultrasonography + magnetic resonance imaging did not present increased sensitivity (22.2%) that went beyond the agreement of the sensitivity found for magnetic resonance imaging + mammography or magnetic resonance imaging + ultrasonography, even though the specificity (93.9%) was higher than the specificity found for magnetic resonance imaging + ultrasonography or ultrasonography + mammography. However, such specificity did not prove to be greater than for the association of magnetic resonance imaging + mammography, for which the likelihood ratio was the highest (6.33). This was because there were a low number of interpretations in agreement (n = 42), and the true positive, false positive and false negative values were distributed evenly.

Thus, mammography should be the examination of choice for patients over 40 years of age who are asymptomatic for rupture. If the implant is considered normal through mammography, it should be borne in mind that the extracapsular rupture type is ruled out. If the implant is considered "young" (i.e. it was included less than five years ago) and if it does not show mammographic alterations such as irregular contours and silicone globules beyond the breast parenchyma, it will probably not be ruptured. The investigation for rupture detection in such asymptomatic patients can then be terminated. On the other hand, if the implant is considered "old" (i.e. it was included more than six years ago), magnetic resonance imaging with a breast coil is the examination of first choice.

If there is a major bulge in "young" implants or the patient has significant anxiety, and mammograms show normal breast parenchyma, magnetic resonance imaging with a breast coil should be performed because of its higher sensitivity.

In patients under 40 years of age, ultrasonography is the method of choice for implant integrity evaluation. If there is any positive ultrasonography sign such as the stepladder sign or echogenic noise, there is no need to perform magnetic resonance imaging. In this case, the implant will very likely be ruptured if it was included more than six to eight years ago. On the other hand, if the "old" implant is anechoic or contains fine internal echoes, it is labeled as non-ruptured and further investigation will not be necessary.

If the implant was included less than five years ago ("young implant") and presents echographic alterations such as thick echogenic lines, echogenic bands, associated or nongrouped amorphous echoes or anterior reverberation, such ultrasound findings are considered to indicate only a suspicion of rupture, or they are non-diagnostic (particularly if the implant is of single lumen silicone gel type). In this case, further investigation by magnetic resonance imaging will be required.

## CONCLUSIONS

From the results obtained from the evaluation of the efficacy of mammography, ultrasonography and magnetic resonance imaging in the detection of breast implant rupture among an asymptomatic population, it can be concluded that magnetic resonance imaging with a dedicated breast coil had the highest sensitivity, while the specificity was similar to the other methods. The statistically significant signs for rupture were the stepladder and snowstorm in ultrasonography and linguine in magnetic resonance imaging. In the evaluation of implant integrity, the presence of envelope folds seen in magnetic resonance imaging was a statistically significant sign.
